# SARS-CoV-2 Risk Management in Clinical Psychiatry: A Few Considerations on How to Deal With an Unrivaled Threat

**DOI:** 10.3389/fpsyt.2020.00550

**Published:** 2020-06-11

**Authors:** Peter M. Kreuzer, Thomas C. Baghai, Rainer Rupprecht, Markus Wittmann, Dagmar Steffling, Michael Ziereis, Marc Zowe, Helmut Hausner, Berthold Langguth

**Affiliations:** ^1^ Department of Psychiatry and Psychotherapy, Bezirksklinikum, University of Regensburg, Regensburg, Germany; ^2^ Clinic of Psychiatry, Psychosomatic Medicine and Psychotherapy, Wöllershof, Germany; ^3^ Emergency Unit, Departments of Neurology and Psychiatry, Bezirksklinikum, University of Regensburg, Regensburg, Germany; ^4^ Public Health Department, Government of the District Upper Palatinate, Regensburg, Germany

**Keywords:** corona virus, SARS-CoV-2, clinical psychiatry, hospital management, pandemic, pandemia

## Abstract

The pandemic spread of the corona virus SARS-CoV-2 has even-handedly shattered national and international health systems and economies almost in an instant. As numbers of infections and COVID-19–related deaths rise from day to day, fears and uncertainties on how to deal with this unknown threat are extremely present both for individuals and societies as a whole. In this manuscript, we aim to exemplarily describe the bullet points concerning (a) the internal risk management, (b) the organizational and structural changes, and (c) the communicational strategies applied in a Psychiatric University Hospital in the Southern part of Germany. The authors are well aware about the fact that almost none of these considerations may be considered as evidence-based at the moment. However, the authors trust that these reflections and experiences may be useful as an orientation for similar risk constellations in other afflicted countries due to the temporal delay of the pandemic course.

## Introduction

The outbreak of the novel coronavirus SARS‐CoV‐2, epicentered in Hubei Province of the People's Republic of China, has affected many other countries worldwide up to now. On January 30, 2020, the WHO Emergency Committee declared the case of a global health emergency (1). On March 11, the WHO made the assessment that COVID-19 can be characterized as a pandemic (2).

In many countries where the virus has spread quickly, medical systems have not been able to keep up with the demand for intensive medical care and mortality rates have been reported high. Italy, in particular, has at least in some regions been overrun by the spread, even with the entire country on lockdown, and the medical system has been overwhelmed, resulting in the need to ration medical care and therefore hazarding many deaths as a consequence (3).

In order to deal with this highly challenging situation, unprecedented measures have been taken. On the societal level the Federal Government of Germany announced (similar like most other countries), an almost complete shutdown of public life with closure of schools, universities, restaurants, shops, etc., with the goal to slow down the spread of SARS-CoV-2. Concerning the health system, all available capacities were reorganized with the goal to provide as many treatment capacities for COVID-19 patients as possible. In particular, the number of beds on intensive care units equipped with ventilation support for COVID-19 patients showing respiratory failure was significantly increased. These two main measures were taken in order to avoid the dangerous mismatch between a sudden extreme need and a limited availability of treatment places on intensive care units.

One further experience from Italy was the particular role of the medical infrastructure regarding the spread of infections (4). With a large proportion of doctors and nurses being infected, the functioning of the health system was severely impaired and there was a high risk for all patients to become infected when they get in contact with the health system (4). However, not only the medical infrastructure but also the lack of protective materials such as surgical masks and FFP masks played an eminent role in the spread of infections.

This situation of enhanced infection risk and scarcity of protective materials poses a complex challenge for every health institution, as several—partly contradictory—goals have to be achieved: First, control the infection rate within the institution, both for patients and staff; second, try to avoid all hospitalizations, that are not extremely urgent; third, provide all available resources for supporting and empowering the intensive care units and fourth, take reasonably care for all other “non-COVID-19” patients. These goals have also been requested by the Federal Government of the Republic of Germany. The contradictory character of these goals is best illustrated by the general decision on how to allocate the resources on hand: an almost complete stop of admissions of patients would provide an excellent strategy for maximized infection control. However, the majority of patients would be left unattended rising the risk of exacerbations of medical conditions on a large scale. Good anti-infection strategies on intensive care units require an enormous amount of protection materials and testing capacities, but how could this be achieved in the context of a general scarcity of supplies and capacities?

In psychiatric hospitals, the situation is particularly demanding. Even less than other medical fields, psychiatry units are not set up for aggressive infection control, staff and patients are not used to wear protective gear, and a great proportion of people with psychiatric illnesses is usually treated on an ambulatory basis. The “treatment as usual” of psychiatric conditions involves intense social interactions which are usually performed with certain physical contact: patients attend therapeutic groups and occupational therapy sessions; they are used to dine in communal areas, watch television, and play games together in day rooms. Patients who are very ill with psychiatric disorders may resist hygiene measures, and they may intrude into the personal space of others. This is well in line with the experiences made in retirement homes where infection and death rates exponentially increased during the course of the pandemic. Parallels regarding the treatment of dementia patients can easily be drawn and were registered in our own experience.

Patients on an acute psychiatric unit may be agitated, uncooperative, or even violent, and it's not hard to imagine the distress of anyone who has a patient spit on them as we're all trying to remember not to shake hands. Moreover, a large proportion of hospital admissions in psychiatry occur as emergencies without any possibility to postpone the hospital stay. With almost all community-based treatment offers and outpatient clinics closed and a situation full of uncertainty and distress combined with social isolation, one also has to consider that the need for inpatient treatment of psychiatric patients developing an acute crisis may even increase.

To provide adequate therapy in the context of a highly contagious pandemic spread requires not only experienced personnel but also adequate spatial, financial, and material resources. Without any doubt, the SARS-CoV-2 pandemic represents a new challenge for psychiatric health care (3).

## Methods

In the present manuscript, we provide information about the measures taken, their feasibility, and the related experiences in the Psychiatric District Hospital of Regensburg located in the Southern part of Germany which also serves as the Department of Psychiatry and Psychotherapy of the University of Regensburg. The hospital provides 525 beds for inpatient treatment in all fields of clinical psychiatry including geriatrics and addiction medicine, a day clinic with 50 places and a large out-patient department. The Department of Psychiatry and Psychotherapy of the University of Regensburg serves a population of nearly 700,000 people as exclusive, single provider of inpatient psychiatric treatment. The hospital is run by a public enterprise (Medizinische Einrichtungen des Bezirks Oberpfalz; medbo) with more than 3,600 employees. Further institutions of the public enterprise include psychiatric hospitals in Wöllershof (distance: 95 km/59 miles) and Cham (60 km/37 miles), additional out-patient facilities in Amberg (70 km/43 miles) and Parsberg (45 km/27 miles), hospitals for forensic psychiatry, child and adolescent psychiatry, neurology, neurological rehabilitation, and residential homes for psychiatric patients.

Affected occupational groups included medical doctors, psychologists, nurses, social workers, music therapists, sport therapists, occupational therapists/ergotherapists, educationalists, and a great variety of technical and administrative supporters (IT, pharmacy, distribution, logistics, purchasing department, infrastructure, carpenters, kitchen staff, etc.).

No ethics approval was necessary for the considerations presented in this manuscript due to the fact that they exclusively rely on theoretical considerations and practical lessons learnt during the early stages of the 2020 SARS-CoV-2 pandemic.

## Results

The authors consider several items as important for the management of the SARS-CoV-2 threat in the context of a psychiatric hospital.

**Figure 1:**
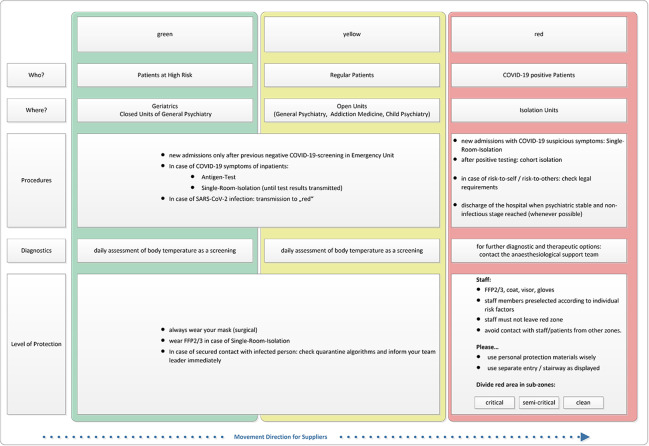
“Traffic light” zone concept.

1. **Leading structure:** A “corona core team” (CCT) involving representatives of all medical and infrastructural fields of the health service provider was established in an early stage of the pandemic. The challenge to deal best with the SARS-CoV-2 pandemic can only be met, if all clinical and non-clinical departments join to work in close cooperation and are enabled to make necessary adjustments to the plans on a daily base.
  In our case, the CCT included the leading physicians and nurses of all hospitals of the enterprise (including the fields of neurology, neurological rehabilitation medicine, general psychiatry, geriatric medicine, addiction medicine, child psychiatry, and forensic psychiatry) and representatives of the hospital management. Moreover, representatives of pharmacy, the hospital blood lab, human resources, public relations, logistics, dispensary, IT and facility management, company medical officers, emergency room administration, and hygienics were involved from the start resulting in regular participation of 35–55 people. At least one representative of each department (either medical or nursing) was obligatory. The participation of all these participants turned out to be extremely important, as all decisions required sufficient knowledge about the current situation from all involved perspectives. As an example, regulations concerning the use of protective gear required detailed information about current availability, expected deliveries of new material, possibilities for re-use, etc. The team was leaded by the CMO (chief medical officer) of the district region by direct order of the president of the health service provider. The team met regularly on a daily basis, initially every workday, later in the course 7 days a week. Right from the start, no meetings were performed on a personal level due to the requirements of physical distancing, all communication was conducted *via* “zoom” web conferencing.
2. **Early shutdown of out-patient treatment facilities and reduction of the number of inpatients:** Outpatient treatments were completely closed down at a very early stage (at infection rate still 0). The shut-down involved both the ambulatory/day clinic (50 patients with 8 h of daily treatment) and the large outpatient clinic (> 8,000 patients/year). Nevertheless, patients were offered close-meshed phone contacts and web conferencing with their therapists. Prescription logistics and urgent treatments (such as intramuscular administration of long acting antipsychotics, regularly drawing blood under clozapine treatment, etc.) were nevertheless provided as accustomed to. According to a related consensus statement (5) intervals of blood tests for patients under clozapine treatment were prolongated. In addition, a patient-centered blog “stay-at-home” (https://www.medbo.de/bleibzuhause/) was launched providing therapeutic input of different fields (psychotherapy, sports, occupational therapy, …) twice a day (9:00 am and 3:00 pm), which could only be retrieved in a time frame of 1 h (in order to provide additional motivation to stay at home and keep in touch with familiar therapists). Moreover, the number of admitted inpatients was reduced as far as possible to gain resources for patient isolation and for transferring staff where necessary. Usually, the inpatient facilities of the hospital are occupied around 97% all over the year. The occupancy was reduced to approximately 60% to provide the necessary flexibility for an effective crisis management.
3. **Early shutdown of cross-sectoral facilities and activities:** The CCT decided to shut down all common facilities such as sporting areas, fitness facilities, occupational therapies/music therapies, etc., involving patients from more than one single ward at a very early stage (at infection rate still 0) to avoid spreading of infections across ward structures. This shut down involved the staff's canteen providing more than 500 hundred daily meals as well. In addition, no business trips were any longer authorized at all. Returning staff from holidays was obliged to contact the company's medical officers (on the phone or *via* email) before entering the hospital's area and re-starting work (especially when returning from Italy's regions “at risk”). Staff members were not allowed any secondary employment any longer and were offered to increase the number of working hours in the hospital to avoid financial damages.
4. **Early and consistent hygiene instructions of both staff and patients:** Patients and staff were instructed right from the start to follow basic physical distancing routines with at least 1.5 to 2 m distance whenever and wherever possible and to completely avoid shaking hands. Patients were instructed to check vital parameters such as routine blood pressure and heart rate measurements by themselves under staff observation to maintain physical distance. Physical examinations and medical procedures such as drawing blood and doing ECG controls were changed from a “conducting as a routine”-level to “conducting when explicitly ordered by doctor”-level. Therapeutic group interventions (on ward-level) and dining rooms were further allowed under requirement of reduced participant number and at least one empty chair between the attendees. Occupancy of inpatient resources were lowered from 100% level to 80% (before first infection in hospital) and later to a 50% benchmark (to be able to consolidate two wards as a new one and unleash staff resources for isolation zones and compensate for quarantine-associated “gaps”). This reduced occupancy led to the possibility to close common rooms and offer patients single room facilities wherever possible. Clinical visits (usually in patients' rooms) were changed to interdisciplinary conversations with the patient in a therapy room with regulations of physical distancing. A big problem in many psychiatric hospitals is that many patients are heavy smokers. Even before the pandemic, patients had to leave the wards to be allowed to smoke cigarettes; this rule was specified including physical distance rules in order to reduce infection risks.
  A difficult challenge was the uncertainty about the availability of protection material. At the daily CCT meetings, the exact instructions for the use of face masks and other protective gear for the different units were discussed depending on the urgency of the need and the current and expected future availability. When available on a large-scale patients and staff members were instructed to continuously wear surgical masks. In one case, six staff members of a single ward became infected (probably due to common dining in their break). However, probably due to the consistent use of surgical masks both for patients and team members no patients became infected in this situation.
  Moreover, hygienic measures (such as cleaning and disinfection of door handles) were redefined following an intensified schedule.
5. **Internal communication and conferencing:** All conferences across locations and hospital departments and even patient consultations where possible were changed to video conferencing to avoid cross-sectional infections. All staff members not urgently needed on the ground were encouraged to work in home office (e.g., almost the entire billing and administration department). The IT department was ordered to provide home office opportunities in a “fast track approval” manner upon request of the particular team leaders.
6. **Staff members at risk:** Already at zero-infections-stage all members of medical professions (mainly medical doctors, nurses, social workers, and psychologists) were contacted by email and asked the following questions: a) Do you have already scheduled holiday plans in the next months? b) Do you need free time to care for your children in case of school lock down? c) Do you need to be employed in a protected area without patient contact (such as telephone counseling) due to any health issue (such as immune deficit, heart/lung/liver diseases, diabetes, or pregnancy)? d) Have you previously worked in an intensive care unit (if yes, how long)? and would you be willing to provide service in such an environment in case of urgent need?
  All data were assessed in a single table with restricted access to the CMO and delegates due to (individual, health-related) data protection regulations. Pregnant colleagues were immediately transferred to home office and telephone counseling workplaces as no reliable data on mid- and long-term outcomes were available at that time point. This was considered a major contribution on how to deal with individual worries and fears concerning the impact of the pandemic on our staff's lives. Many members of our personnel were not only worried by individual comorbidities and risk factors (such as age, etc.) but also by a putative impact of an infection on the lives of family members at risk. In case of potential (or documented) infection of staff members home quarantine measures were carried out thoroughly and the rest of the team was tested (at least when sufficient testing capacities were available).
7. **Early outgoing and visit restrictions:** On March 25, 2020, the State of Bavaria (Germany) declared the state of emergency and announced outgoing restrictions only allowing to leave home only in case of (a) receiving medical care, (b) shopping daily care items, (c) assisting others in need, (d) going to work, and (e) doing sports on individual level or with family members of the same household. Already one week before, all patients had been informed that they were not allowed to leave the area of the hospital (especially not to travel by bus or train) and that visitors were no longer allowed to enter the wards and could only meet their family members on a walk around the hospital's parks. Visitors were completely prohibited to enter the area of the hospital in case of acute illness (especially in case of common cold symptoms), having traveled to an “area at risk” (by classification of federal institutions) or having had contact to a confirmed COVID-19-case. Before and during the pandemic, acute patients (suicidal or dangerous) were legally involuntarily admitted to our hospital: some of the judges in charge continued to visit these patients (after a thorough explanation of infection protection requirements), others decided to communicate with these patients *via* web conferencing or telephone.
8. **Screening procedures and admittance strategies:** All *newly admitted* patients were regarded as “potentially infectious”. A web-based pre-screening procedure has been established. Patients who contact the hospital with the intention to get admitted are advised to complete a web-based questionnaire that asks about the main complaint and includes also a few screening questions about their COVID-19 risk. The answers of the patients are visible for the medical doctors at the admittance unit and enable them to call the patient, to discuss whether the hospital admission is necessary or whether there exist alternatives. Moreover, a screening unit was established in the entrance of the hospital, which has to be passed by all patients before entering the emergency and admission department. The screening procedure involves questions concerning risk behavior, travel history, contact with infected persons, COVID-19-symptoms, and common cold symptoms. Body temperature is measured, and a SARS-CoV-2 PCR test is performed (starting April 9^th^, as soon as sufficient test capacities were available). Patients are only admitted to the hospital after careful consideration of the indication for in-patient treatment taking into account the potentially increased infection risk in the hospital. Therefore, a senior physician is involved in every single admittance case to assure that the psychiatric condition can only be managed by in-patient stay and no intensified “remote” therapeutic offers such as changes in medication or frequent phone calls/web conferencing. Patients with suspicion for SARS-CoV-2 infection are directly admitted to the psychiatric isolation units, all other patients are admitted into “admittance single rooms” at each of the wards (open and closed) in a diagnosis-specific manner for 24–48 h of “single-room-isolation as a precaution” with regular assessment of body temperature and (if available depending on capacities) SARS-CoV-2 PCR testing.
*All patients at all wards* are assessed body temperature as a screening procedure once daily and instructed to immediately report any (even unspecific) symptoms of common cold or diarrhea (6). In addition, strategies to test for anosmia were clinically tested according to prior work by Russell (7) and Lechien (8).
9. **Establishment of isolation facilities and a “traffic light zone concept”:** According to rising infection rates, one (formerly open) ward was transferred to an “isolation unit” with 12 rooms for the treatment of COVID-19-positive patients with psychiatric diseases. This was communicated to local authorities as part of an emergency case concept. The isolation unit was separated in a unit for cohort isolation (for patients with confirmed COVID-19) and a unit for single isolation (for patients who were considered at risk for SARS-CoV-2 infection at hospital admission and for patients who were close contact persons with confirmed COVID-19 patients). In the course of the pandemic, it turned out, that “contact individuals” (with ≥ 15 min of cumulative contact and a contact distance ≤ 2m) that needed quarantine of 14 days incubation time and were not dischargeable for quarantine at home occupied many of these resources. Moreover, for the patients at risk of infection, it took several days to rule out a potential SARS-CoV-2 infection, as a single negative test was not considered reliable enough. Therefore, even with few confirmed COVID-19 patients, there was an increasing demand for single isolation capacities. Therefore, a second ward with additional ten rooms was turned into a further “isolation unit” and the doctors' team of both wards formed an “isolation team” taking care for both neighboring wards covering 8:30 am to 8:00 pm 7 days a week. The members of this “isolation team” were released from “doctor on duty” shifts in charge of the whole rest of the hospital due to infection protection regulations.
  In addition, a “traffic light” zone concept was established: the concept was shaped to instruct all comprehensive service providers (e.g., property cleaning, catering, supply of materials, consultants of other medical fields, …) to move from green to yellow to red zone. The “green zone” was labeled as “sensitive” due to the “patients at risk” treated there (e.g., elderly patients in geriatrics), the “yellow zone” consisted of the “regular patients” and the “red zone” was formed by the isolation units described already above. **Figure 1** illustrates further details of each zone.
10. **Test as much as possible:** The SARS-CoV-2 pandemic is characterized by a great uncertainty about the infection status of an individual together with a high contagious potential of infected individuals (possibly without any clinical symptoms). At the current stage, only PCR antigen tests are available as established lab-tests to confirm the infection in an individual, but also these tests have a considerably high false-positive and false-negative risk (9). Moreover, the availability of these tests was limited at the start of the pandemic, resulting in long delays between testing and information about results. Despite all the uncertainties with PCR tests, we aimed at trying to test as many patients and staff as possible, to be able to make informed decisions. From the beginning, all patients and staff with symptoms suggestive of a SARS-CoV-2 infection were tested. In case of a positive test, all patients and the whole staff of the ward were tested. With increasing availability of test capacities, each patient was tested at admission and a system of regular tests in asymptomatic staff members was established. In order to take the possibility of false positive and false negative tests into account, we tried to perform multiple tests in symptomatic individuals. Moreover, we tried to make use of all available clinical information. As sudden loss of taste and smell is a frequently reported symptom (7, 8), we established olfactory tests as an additional screening tool. Moreover, in order to increase testing capacities, resources normally dedicated to research were utilized for routine laboratory testing. To date a total of 67 tests turned out and were confirmed as positive (both for staff and patients).
11. **Effects on teaching:** All teaching activities (for medical students as well as nursery training attendees) have to be provided by means of video systems feasible for lectures and seminars. Most of our teaching activities were conducted by means of zoom and moodle. A particular difficulty is how to deal with bedside teaching in small groups which is regarded an important feature in the training of psychiatric skills. It has—when writing these lines—yet to be decided by the authorities to what extent bedside teaching in medical training can be replaced by online courses according to pandemic exit strategies and legal issues as well.
12. **Effects on research:** The outbreak of the SARS-CoV-2 crisis exerted a dramatic impact on research activity both in basic science and clinical research. With regard to clinical science all research activities enrolling patients and volunteers in research studies had to be interrupted to minimize infection risks. On the other hand, clinical research staff resources had to be dedicated to patient care which was also reinforced by the government. In addition, both human and laboratory resources for basic sciences were used to support laboratory testing to optimize clinical management. It remains to be determined when research can restart successfully in respective pandemic exit strategies.
13. **Communication with regional health care authorities**: Official regulations concerning the management of the SARS-CoV-2 pandemic have regularly been issued by the official Health Authority in Germany (Robert-Koch-Institut; RKI) and the State Government of Bavaria (Landesamt für Gesundheit und Lebensmittelsicherheit; LGL). However, their exact interpretation and the transfer to our hospital's specific situation required close communication with the local authorities. Local health care authorities are by law authorized to control hygiene measures in each hospital of the district and have a right to require beds in case of severe urgency. An overall web-based platform was set up including all hospitals of the entire State of Bavaria where free beds and capacities for intensive care are to be communicated on a daily basis.
  As the local health care authorities were also overwhelmed with all their duties in the pandemic situation, we were glad about a responsive “fast track” contact with the responsible medical doctor of the district government.
14. **Transparent communicational strategies concerning both patients and colleagues:** As at least one member of the public relations department of our hospital took part in the daily meetings of the CCT a transparent and fast communication strategy *via* the intranet news feed or smartphone applications with daily updates (for internal information of the staff) and our public homepage (for advice on how to deal with the pandemic in case of seeking help in our hospital) was ensured. It turned out that it was necessary and regarded as helpful to frankly report infection rates among patients and staff and to communicate “hot spots” without any delay in order to preserve the trust of the employees in the management of the situation. This was even more the case because many of the head physicians had to avoid regular visits on all of the wards they were in charge of due to infection protection reasons. Podcasts and notifications of the president of our hospital turned out to be more than useful to maintain good spirits among the different staff members.

## Discussion

The self-efficacy of human beings seems to strongly rely on the stability of the notion that based on our *yesterday's experience* we trust that we can strongly rely on our *today's condition* and predict the environmental *changes of tomorrow*. This notion is strongly disrupted in the actual SARS-CoV-2–related crisis due to the exponential rise of infection rates challenging limited hospital capacities, the unstable supply of personal protection items and the fast evolving official recommendations/regulations that have to be taken into account when establishing risk management concepts in the context of psychiatric hospital care. The authors are convinced, that the dynamic character of this situation is the biggest challenge concerning communicational strategies both with patients and colleagues.

The need for additional labor power, the shift of colleagues experienced in intensive care procedures to other medical fields, the gap of colleagues “at risk” or “under quarantine” into home office and protected working spaces, the need for organizational and structural changes (such as “isolation units”), and the integration of colleagues of other areas of expertise into well-rehearsed teams require an enormous amount of management capacities. These challenges must be taken into account as early as possible in order to provide sufficient personnel and spatial resources for these tasks. However, some pitfalls emerged and should not be concealed: one major issue (causing many disturbances) was that even after thorough discussions in the CCT, it was a challenge to provide specific information for all involved parties and maintain reliable information channels for weeks up to now. For example, many of the measures mentioned above required an intensified collaboration between members of administration, different occupational groups of the hospital, infrastructure, supply, IT, and legislation. Additionally, in the first phase of the pandemic, most colleagues were not allowed holiday leaves causing exhaustions on an individual and team level as well as administrative concerns due to the fact the huge amounts of holiday claims were postponed.

For almost all staff members, the current situation requires massive alterations from their daily routine. As the pandemic situation in general, the infection rates in the hospital, the availability of protective material as well as the public regulations are unpredictably changing on a day-to-day level, there is a strong need for frequent adjustments of the internal organization. It turned out, that our hospital's “step-wise emergency plan” in case of catastrophes was completely overrun by the dynamic of this pandemic. For many doctors and nurses this means that they have to change their working place within the hospital and take over new tasks. This usually happens with very short notice. Our experience is that the majority of our colleagues found a way to deal with this situation. However, some struggle hard when having to transfer their areas of expertise (such as advanced psychotherapeutic skills) to other settings (for example, the need for substantial knowledge of legal requirements in involuntary treatments). Moreover, many of our colleagues were not only worried by the potential impact of an infection on their own health state but expressed intense fear that they might be responsible for infections of family members at risk. The most important feedback from these colleagues was that a key requirement in this situation is clear, transparent, and reliable communication. In the meantime, centralized information on how to deal with the strain of working in a potential infectious surrounding and the possibility of social isolation due to quarantine is made available, e.g., *via* https://www.upd.ch/de/forschung/psychiatrische-rehabilitation/pandemiebewaeltigung-psychiatrie.php.

Regarding our patients, the requirements of “remote psychiatric care” drive the field forward requiring a fast implementation of new technologies (patient-centered blogs, individualized web-based psychotherapy) in our daily routines allowing therapists to provide psychiatric care from home office work spaces directly to connected patients. The medical field of psychiatry seems (in our own experience) to be more suitable for these new attempts than any other medical field because the assessment of psychopathological issues and the provision of therapy strongly rely on communication that can (at least on a temporary level) be provided in a remote way. However, a “core” of psychiatric patients *does* require immediate personal attendance and intensified in-patient treatment without any chance of temporal delay for example in case of acute psychotic symptoms with strong misjudgments of reality or suicidal ideations.

Many discussions took place in our institution on how our patients suffering from (in most cases) severe psychiatric conditions might manage to cope with the variety of restrictions attributed to the SARS-CoV-2 situation. However, it turned out that the vast majority of psychiatric patients is evidently very able to deal with fears and uncertainties (in many cases even exceeding the capability of the rest of the population). The authors are constantly surprised by the extensive amount of understanding and support they receive from their patients every day. However, at the time of writing this manuscript, we have only the experience of 4 weeks of closure of the outpatient clinic and 3 weeks of public shut-down. It is possible that with longer duration of the situation, more and more people will require support beyond phone or web conferencing consultation. This might be the case both for people with chronic psychiatric diseases and those, who develop psychiatric problems for the first time in their lives as a reaction to the burden caused by the pandemic.

All of the measures mentioned above were taken in view of an uncertainty of the economic situation. Of course, the shutdown of ambulatory and day clinics and the reduction of the number of inpatients in view of the necessity of additional resources for infection protection is a great economic burden. On the other hand, the Federal Government has promised to provide additional resources for hospitals adapting for crisis management including psychiatric hospitals with payments of 560 Euro per “lost inpatient day” in comparison to the year 2019 (since March 16, 2020).

The authors are well aware about the preliminary nature of all the considerations mentioned above and do not a bit want to give the impression “to know better” at all. However, we decided to publish these considerations that were followed in order to prepare our psychiatric hospital for a (hopefully) successful management of this crisis. We therefore hope to enable others in similar situations to avoid some of the lessons that we had to learn so far. We are not able and will never be able to judge the efficacy of the above mentioned measures, but this is the case for many preventive procedures that must be taken at the moment. Moreover, we are well aware that the situation of our hospital might differ from many other psychiatric hospitals, especially since the context is highly relevant for all taken measures and we—when writing these lines—have had only three weeks of experience with this situation.

Nevertheless, we can report, which measures have proven to be feasible in a large psychiatric hospital and which early experiences we have gathered with them. Our most important lesson is the enormous importance of an early establishment of reliable and transparent communicational strategies allowing to keep in touch with both patients and colleagues.

## Data Availability Statement

The original contributions presented in the study are included in the article/supplementary material; further inquiries can be directed to the corresponding author.

## Author Contributions

All authors contributed to the article and approved the submitted version.

## Funding

Open Access publication fees have been granted by the University of Regensburg.

## Conflict of Interest

The authors declare that the research was conducted in the absence of any commercial or financial relationships that could be construed as a potential conflict of interest.

## References

[1] Velavan TP Meyer CG The COVID-19 epidemic. Trop Med Int Health : TM IH (2020) 25(3):278–80. 10.1111/tmi.13383 PMC7169770 32052514

[2] Buoro S Di Marco F Rizzi M Fabretti F Lorini FL Cesa S Papa Giovanni XXIII Bergamo Hospital at the time of the COVID-19 outbreak: letter from the warfront. Int J Lab Hematol (2020). 10.1111/ijlh.13207 32222091

[3] Miller D Coronavirus on the Inpatient Unit: A New Challenge for Psychiatry - Medscape - Mar 16, 2020. 2020 [11.04.2020]. Available from: https://www.medscape.com/viewarticle/926834?src=wnl_tp10n_200410_mscpedit&uac=105181FG&impID=2340632&faf=1#vp_1.

[4] Rosenbaum L Facing Covid-19 in Italy - Ethics, Logistics, and Therapeutics on the Epidemic's Front Line. N Engl J Med (2020) 382(20):1873–5. 10.1056/NEJMp2005492 32187459

[5] Siskind D Honer WG Clark S Correll CU Hasan A Howes O Consensus statement on the use of clozapine during the COVID-19 pandemic. J Psychiatry Neurosci : JPN (2020) 45(4):200061. 10.1503/jpn.200061 32297722 PMC7828973

[6] Li XY Dai WJ Wu SN Yang XZ Wang HG The occurrence of diarrhea in COVID-19 patients. Clin Res Hepatol Gastroenterol (2020) S2210-7401(20):30092–9. 10.1016/j.clinre.2020.03.017 PMC7270575 32253163

[7] Russell B Moss C Rigg A Hopkins C Papa S Van Hemelrijck M Anosmia and ageusia are emerging as symptoms in patients with COVID-19: What does the current evidence say? Ecancermedicalscience (2020) 14:ed98. 10.3332/ecancer.2020.ed98 32269598 PMC7134577

[8] Lechien JR Chiesa-Estomba CM De Siati DR Horoi M Le Bon SD Rodriguez A Olfactory and gustatory dysfunctions as a clinical presentation of mild-to-moderate forms of the coronavirus disease (COVID-19): a multicenter European study. Eur Arch Otorhinolaryngol (2020) 1–11. 10.1007/s00405-020-05965-1 32253535 PMC7134551

[9] Zhuang GH Shen MW Zeng LX Mi BB Chen FY Liu WJ [WITHDRAWN: Potential false-positive rate among the ‘asymptomatic infected individuals' in close contacts of COVID-19 patients]. Zhonghua Liu Xing Bing Xue Za Zhi (2020) 41(4):485–8. 10.3760/cma.j.cn112338-20200221-00144 32133832

